# Highly efficient generation of sheep with a defined *FecB*^*B*^ mutation via adenine base editing

**DOI:** 10.1186/s12711-020-00554-6

**Published:** 2020-07-01

**Authors:** Shiwei Zhou, Yige Ding, Jiao Liu, Yao Liu, Xiaoe Zhao, Guanwei Li, Chenguang Zhang, Chao Li, Ying Wang, Peter Kalds, Yawei Gao, Bo Zong, Xiaoyu Huang, Shuhong Huang, Honghao Yu, Qifang Kou, Bjoern Petersen, Xingxu Huang, Xiaolong Wang, Baohua Ma, Yulin Chen

**Affiliations:** 1 Key Laboratory of Animal Genetics, Breeding and Reproduction of Shaanxi Province, College of Animal Science and Technology, Northwest A&F University, Yangling, China; 2 College of Veterinary Medicine, Northwest A&F University, Yangling, China; 3 College of Biotechnology, Guilin Medical University, Guilin, China; 4 Ningxia Tianyuan Tan Sheep Farm, Hongsibu, China; 5 Institute of Farm Animal Genetics, Friedrich-Loeffler-Institut, Neustadt, Germany; 6 School of Life Science and Technology, ShanghaiTech University, Shanghai, China

## Abstract

Base editing has the potential to improve important economic traits in agriculture and can precisely convert single nucleotides in DNA or RNA sequences into minimal double-strand DNA breaks (DSB). Adenine base editors (ABE) have recently emerged as a base editing tool for the conversion of targeted A:T to G:C, but have not yet been used in sheep. ABEmax is one of the latest versions of ABE, which consists of a catalytically-impaired nuclease and a laboratory-evolved DNA-adenosine deaminase. The Booroola fecundity (*FecB*^*B*^) mutation (g.A746G, p.Q249R) in the *bone morphogenetic protein receptor 1B* (*BMPR1B*) gene influences fecundity in many sheep breeds. In this study, by using ABEmax we successfully obtained lambs with defined point mutations that result in an amino acid substitution (p.Gln249Arg). The efficiency of the defined point mutations was 75% in newborn lambs, since six lambs were heterozygous at the *FecB*^*B*^ mutation site (g.A746G, p.Q249R), and two lambs were wild-type. We did not detect off-target mutations in the eight edited lambs. Here, we report the validation of the first gene-edited sheep generated by ABE and highlight its potential to improve economically important traits in livestock.

## Introduction

Clustered regularly interspaced short palindromic repeat (CRISPR)/CRISPR-associated (Cas) 9 has been widely used to produce gene-edited animals and plants [1]. Recently, the CRISPR/Cas9 system has emerged as a simple, rapid and precise editing tool in the genomes of livestock. However, the application of this system for the introduction of defined point mutations requires the generation of double-strand DNA breaks (DSB) under the guidance of a single guide RNA (sgRNA) and homology recombination (HR) via single-stranded oligodeoxynucleotides (ssODN). Non-homologous end-joining (NHEJ) and HR are the two major DSB repair pathways [2–5]. The NHEJ pathway is active throughout the entire cell cycle and is more efficient in the repair of DSB. Thus, it is inefficient to generate animal models with defined point mutations via the HR method [6]. The base editing systems emerged thanks to the engineering of CRISPR/Cas9. Currently, two major classes of base editors exist, cytosine base editors (CBE) and adenine base editors (ABE) [7, 8].

The original CBE were developed to convert targeted C:G to T:A [7, 9] and comprised four molecules: a cytosine deaminase that catalyzes the conversion of C to U; a modified Cas9 (nCas9/dCas9) that binds target DNA; an sgRNA that directs Cas9-cytosine deaminase to the target locus; and a UGI that subverts the cellular uracil base excision repair (BER) pathway [10]. After several generations of optimization, base editor 3 (BE3) was developed and applied in a wide range of organisms, with mutation efficiencies higher than 50% in mammalian cells, mouse embryos, tripronuclear human embryos, rabbits, sheep, and goats [7, 11–16]. Since in farm animals, many of the economic traits are due to A:T to G:C substitutions, a catalytically-impaired nuclease has been fused with a laboratory evolved DNA-adenosine deaminase termed ABE to convert targeted A:T to G:C [8]. ABE catalyzes the deamination of adenine to inosine, which is treated as guanosine by the polymerase. Following DNA replication, the A:T base pairs are converted to G:C base pairs. ABE of the *E. coli* tRNA adenosine deaminase (TadA) have evolved through mutations, resulting in the formation of four commonly used ABE, namely ABE6.3, ABE7.8, ABE7.9, and ABE7.10 [17] with ABE7.10 being the most active base editor that shows an average editing efficiency of up to 53% [10]. The editing window of ABE7.10 targets adenosine at the protospacer adjacent motif (PAM) position 4–7 (PAM counted as 21–23), while the other three versions have slightly wider editing windows at position 4–9. However, the editing efficiency may be lower at position 4–9. To further improve the efficiency of ABE7.10, ABEmax and xCas9-ABE were generated separately. ABE7.10 was optimized through the modification of nuclear localization signals (NLS) and codon usage to obtain ABEmax. The resulting ABEmax editor can alter single nucleotide polymorphisms (SNPs) with substantially increased efficiency and low genome-wide off-target activity in a variety of mammalian cell types [18]. xCas93.7 replaced the SpCas9 of ABE7.10 to obtain xCas9-ABE, which leads to higher base editing efficiencies than ABE7.10 in HEK293T cells [19]. Although cytosine base editors often produce cell populations with mixed base editing populations, ABE do not prominently show A to non-G substitutions at the target sites. ABE also have advantages over other approaches in terms of off-target effects. In comparison with Cas9, ABE have a low off-target rate [10]. To date, the application of ABE has been validated in various organisms including human embryos, mice, rabbits, zebrafish, rice, wheat, *Arabidopsis*, and *Brassica napus* [13, 20–25].

The *bone morphogenetic protein receptor 1B* (*BMPRIB*) gene was first identified in Booroola merino sheep, and was shown to be a major factor associated with increased ovulation rates. This gene influences follicular granulosa cell differentiation and follicular development, thus promoting ovulation [26–30]. The *FecB*^*B*^ mutation (g.A746G, p.Q249R) in *BMPR1B* is highly associated with increased ovulation rate and litter size in domestic sheep breeds [28, 31–37]. In previous studies, we used the BE3 system to induce a p.R96C mutation in the sheep *suppressor of cytokine signaling 2* (*SOCS2*) gene and nonsense mutations in the goat *fibroblast growth factor 5* (*FGF5*) gene. The results demonstrated that CBE could be used to induce single base substitutions (C > T) in large animals with a high efficiency [15, 16]. However, to date, the application of ABE for the generation of genetically-edited large animals has not been reported. In this study, we used ABE (ABEmax) to introduce the *FecB*^*B*^ mutation in the genome of Tan sheep, a Chinese local breed. ABEmax mRNAs and sgRNA were coinjected into ovine one-cell stage zygotes, followed by transfer of the developing embryos into surrogate ewes. Although the gene-edited lambs at the *FecB*^*B*^ site were generated via Cas9:ssODN, the editing efficiency was low (22.7%) [33]. In the present study, by using ABE to generate founders with defined point mutations in the *BMPR1B* gene, we observed a much higher editing efficiency compared with the conventional ssODN approach. These results highlight the feasibility of base editors, including both CBE and ABE, to generate large animal models with targeted single nucleotide substitutions.

## Methods

### Animals

All experimental animals were raised at the Ningxia Tianyuan Sheep Farm, Hongsibu, Ningxia Autonomous Region, China. Water and standard food were provided ad libitum. Animals were treated according to the Guidelines for the Care and Use of Laboratory Animals at Northwest A&F University.

### Design of sgRNA

The sequences targeting the g.A746G (p.Q249R) mutation in the ovine *BMPR1B* gene are listed in Additional file 1: Table S1. Two oligonucleotides (see Additional file 1: Table S2) were used for the in vitro transcription of sgRNAs, which were then synthesized and annealed to form double-stranded oligos. These oligos were subcloned into the pUC57-T7-gRNA vector as previously described [38]. The clones that contained the desired sequences were selected, expanded in culture, and the plasmids were extracted using a plasmid extraction kit (AP-MN-P-250G; Axygen, Union City, CA, USA). The sgRNAs were in vitro transcribed using the MEGAshortscript Kit (AM1354; Ambion, Foster City, CA, USA) and purified using the MEGAClear Kit (AM1908; Ambion). Subsequently, the ABEmax in vitro transcription vectors were used as templates to produce ABE mRNAs following previously published protocols [38].

### Screening for high-efficiency ABE versions in sheep fibroblasts

Tissues from a 40-day-old sheep fetus were sectioned and cultured in DMEM medium (Gibco) containing 10% fetal bovine serum (FBS) (Gibco) and 1% penicillin–streptomycin (Gibco). After 3 to 5 days of culture, fetal fibroblasts were isolated and cultured until 70 to 90% confluence. Transfections were performed as previously reported [39]. Briefly, sheep fetal fibroblasts were respectively transfected with *FecB*^*B*^ sgRNA (2.5 μg/μL) and ABE (ABE7.10, ABEmax and xCas9-ABE) plasmid (5 μg/μL) using Lipofectamine 3000 Reagent (Invitrogen) in 6-well culture plates. Forty-eight hours post-transfection, 0.2 μL of puromycin (10 μg/μL) was added to the medium and cells were cultured for 36 h. Then, the culture medium was replaced with puromycin-free medium to permit the complete growth of fetal fibroblasts. Genomic DNA was extracted from transfected and drug-screened fibroblasts and used for Sanger sequencing and targeted deep-sequencing. The list of primers is in Additional file 1: Table S3.

### Production of mutated sheep

Ten healthy ewes (3 to 5 years old) with normal estrous cycles were selected as donors for zygote collection. The superovulation treatment of the donors was performed as previously described [40]. Briefly, an EAZI-BREED controlled internal drug release (CIDR) Sheep and Goat Device (containing 300 mg of progesterone) was inserted into the vagina of the donor ewes for 12 days and superovulation was performed 60 h prior to the removal of the CIDR Device. Each female donor was subjected to natural mating three times, the first mating was carried out 12 h after the initial estrus, and then subsequent matings were performed at 12 h intervals. Zygotes at the 1-cell stage were collected 48 h after the initial estrus by surgical operation and immediately transferred to TCM-199 medium (Gibco, Gaithersburg, MD, USA). ABEmax mRNA (25 ng μL^−1^) and sgRNA (10 ng μL^−1^) were co-injected into the cytoplasm of the collected zygotes using an Eppendorf FemtoJet system [41]. The injection pressure, compensatory pressure, and time parameter were 45 kPa, 7 kPa, and 0.1 s, respectively. Microinjections were performed on the heated stage of an Olympus ON3 micromanipulation system. Injected embryos were cultured in Quinn’s Advantage Cleavage medium (Sage Biopharma, Toronto, Canada) for 24 h and were then transferred into surrogates as previously described [39]. On average, 5.3 embryos were transferred to each recipient. The details on the number of embryos transferred to each recipient and the parents of the embryos are in Additional file 1: Table S4. Pregnancy was confirmed by observing the estrous behaviors of the surrogates at each ovulation cycle. After three estrus cycles (~ 60 days), six of the 18 recipients were pregnant. After around 150 days of pregnancy, six ewes gave birth to eight lambs; no stillbirth or dead animals were found. The eight lambs had good health conditions.

### Genotyping of generated founders

Peripheral venous blood of 2 week-old lambs was sampled to extract genomic DNA. After polymerase chain reaction (PCR) amplification, Sanger sequencing was performed using the KOD-NEO-Plus enzyme (DR010A; TOYOBA, Osaka, Japan). The primers used are in Additional file 1: Table S3.

### Prediction of off-target sites

The potential off-target sites with not more than three mismatches were predicted using the freely available tool Cas-OFFinder [8]. Off-target sites were searched as previously described [8]. The primers for amplifying the off-target sites and Sanger sequencing are in Additional file 1: Table S5.

### Captured deep-sequencing

Target mutations were amplified using a KAPA HiFi HotStart PCR Kit (#KK2501; KAPA Biosystems, Wilmington, MA, USA) to generate deep-sequencing libraries as previously described [16]. The pool of PCR amplicons was sequenced using the MiniSeq with TruSeq HT Dual Index system (Illumina, San Diego, CA, USA).

## Results and discussion

### Screening for high-efficiency ABE versions in sheep fibroblasts

**Fig. 1:**
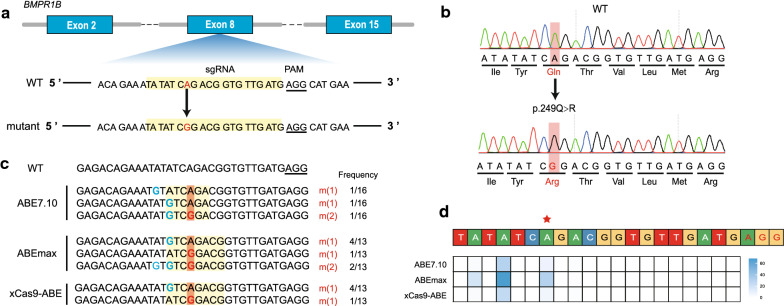
Evaluation of different ABE system mediated nucleotide substitutions of *BMPR1B* in sheep fibroblasts. **a** Schematic view of the target site in the sheep *BMPR1B* gene. sgRNA sequences are displayed in a yellow background. PAM sequences are underlined. The ABE-mediated nucleotide substitutions (g.A746G, p.Q249R) are highlighted in red. **b** Editing efficiency with ABE7.10, ABEmax, and xCas9-ABE in sheep fibroblasts. The editing window are displayed in a yellow background. The ABE-mediated nucleotide substitutions (g.A746G, p.Q249R) are highlighted in an orange background and red. Bystander mutations are indicated in blue. **c** Sanger sequencing chromatogram of intended mutations derived by the ABE system. **d** In sheep fibroblasts, editing efficiency with ABE7.10, ABEmax, and xCas9-ABE through deep sequencing in sheep fibroblasts. Bystander mutations are marked in blue. Three adenine base editors mediated nucleotide substitutions (g.A746G, p.Q249R) are highlighted in red

To obtain edited sheep fetal fibroblast cell lines at the target site (p.Q249R) of the *BMPR1B* gene, we co-transfected sgRNA and ABE (ABE7.10, ABEmax, or xCas9-ABE) plasmids into sheep fetal fibroblasts. The designed sgRNAs encompassed the target point mutation (p.Q249R) in the *BMPR1B* gene (Fig. 1a). DNA from transfected cells was used for Sanger sequencing, which showed overlapping peaks in the sequencing map (see Additional file 2: Figure S1). Then, we further analyzed specific genotypes using TA cloning (Fig. 1b and c). The results showed that the editing efficiency with ABE7.10, ABEmax, and xCas9-ABE plasmids was up to 3/16, 7/13 and 5/13, respectively, whereas the efficiency at the target site (p.Q249R) was 1/16, 3/13 and 1/13, respectively. Next, we carried out targeted deep-sequencing to validate the accuracy of TA cloning and obtained results that were consistent with TA cloning (Fig. 1d) and with those of other recent studies at the cellular level [11, 17, 18]. The efficiency of the ABEmax plasmid was the highest up to 53.8% (7/13), thus it was selected to produce the targeted *FecB*^*B*^ mutation in sheep. These findings highlight how ABE can directly introduce A:T to G:C mutations into sheep fetal fibroblasts, and how it could be a more efficient approach for the generation of gene-edited sheep.

### Generation of edited lambs

To generate lambs with a p.Q249R mutation in the *BMPR1B* gene, we micro-injected sgRNA and ABEmax mRNAs into the cytoplasm of 1-cell stage embryos. Ten mated Tan sheep donors were superovulated and fertilised by natural mating, producing 96 one-cell stage fertilized oocytes [38]. Of the 96 microinjected embryos, 95 were in good condition and transferred into the ampullary-isthmic junction of the oviducts in 18 recipient ewes. Finally, we obtained six pregnancies that reached a full-term gestation period (~ 150 days) and eight lambs (#25, #28, #30, #31, #34, #46, #50, and #52) were born (Table 1). Four ewes (#016, #132, #018, and #708) delivered singletons (#28, #30, #31, and #46, which were not related to each other), and two ewes (#640 and #608) delivered twins (#25 and #34, and #50 and #52, each pair being full-sibs). Details on the lambs’ parents and the recipients are in Additional file 1: Table S4.

**Table 1:** Summary of the sheep obtained with the targeted point mutations via ABEmax

Donor sheep	10
Collected embryos	96
ABEmax-sgRNA	
Injected embryos	96
Transferred embryos	95
Recipient sheep	18
Pregnant recipients	6
Newborns	8
Expected defined substitution	6
Un-defined substitution	2

ABEmax: the latest version of adenine base editors; sgRNA: single guide RNA

**Fig. 2:**
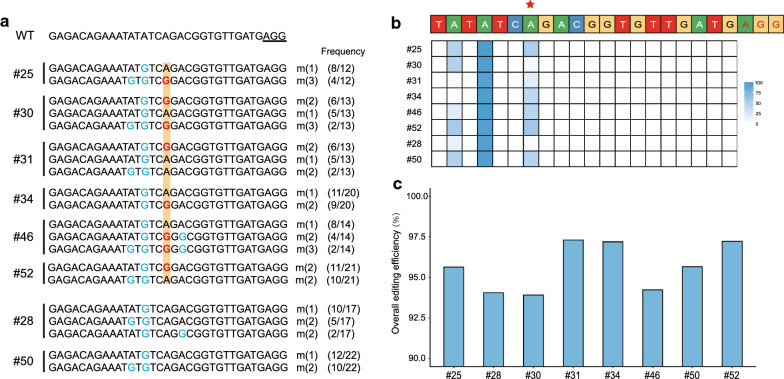
Detection of ABEmax-mediated nucleotide substitutions in founder animals. **a** Genotypes of target sites by TA cloning in all founder animals. Bystander mutations are highlighted in blue. The ABEmax-mediated nucleotide substitutions (g.A746G, p.Q249R) are highlighted in red. **b** Genotypes of target sites through deep sequencing in eight founder animals. Bystander mutations are marked in blue. The ABEmax-mediated nucleotide substitutions (g.A746G, p.Q249R) are highlighted in red. **c** Mutation rate at the targeted region in eight founder animals

We extracted genomic DNA from the blood of these eight lambs and amplified it by PCR-based Sanger sequencing (see Additional file 1: Table S3). Sanger sequencing primarily showed that these eight lambs displayed editing events within the base editing window of ABEmax, but only six of them (#25, #30, #31, #34, #46, and #52) had the specific edit that conferred the desired Q249R substitution (see Additional file 3: Figure S2). To analyze the specific genotypes of each edited lamb, we performed TA cloning to validate single nucleotide substitutions (Fig. 2a). Sanger sequencing confirmed that lambs #25, #30, #31, #34, #46, and #52 were heterozygous mutants at the *FecB*^*B*^ mutation site. The results of TA cloning showed that all the founders had bystander mutations. Three bystander mutations (all are A to G) were detected: from 5′ to PAM direction; A_12_ (Tyr to Cys) in seven founders, A_14_ (Ile to Met) in eight founders, and A_19_ (Thr to Ala) in two founders. In addition, we performed targeted deep sequencing and the results were consistent with those of TA cloning (Fig. 2b and c). All the generated founders were mosaic. This mosaic state is the result of the direct injection of the ABEmax mRNA and sgRNA in one-cell stage embryos. The translation of the injected mRNA occurs at different time points during the division of the embryo, which generates different genotypes in the same individual. In addition, recent studies showed that the non-specificity of ABEmax leads to multiple gene editing outcomes in different tissues of the generated founders [13, 42]. The mosaic state of the founders in the present study is also consistent with our previous gene-editing results [15, 16]. The potential influence of bystander mutations on fertility will be investigated further using the lambing data of the offspring. Efforts have been made to minimize the bystander effects of ABE and improve the DNA specificity [17, 18, 43]. In this study, six out of eight founders were generated with the defined point substitution (Table 1). The efficiency of single base substitutions was significantly higher than in our previous Cas9:ssODN studies on goats (24%) [44] and sheep (22.7%) [33]. In spite of the high efficiency of ABEmax, the wider base editing window at position 2-9 (PAM counted as 21–23) converts all “A” to “G” bases [17]. A reduction of the deaminase activity and the search for new Cas9 proteins are required in order to make ABEmax more precise and increase its range of use. In the future, we anticipate the development of new base editors that show improved targeted DNA specificity and reduce bystanders in the editing window.

### Analysis of off-target mutations in edited animals

To evaluate the off-target effects of ABE, we used Sanger sequencing. Five off-target sites (OT1–OT5) were predicted (see Additional file 1: Table S6) using the Cas-OFFinder program [8]. PCR products from eight gene-edited founders and single gene-edited fetal fibroblasts were sequenced and no off-target events were detected (see Additional file 4: Figure S3 and Additional file 5: Figure S4). Thus, compared to CBE [7, 13, 45–47], ABE produce almost no off-target events [21–24, 42, 48]. These results highlight the accuracy and potential applications of ABE for gene therapy. Although we used the Cas-OFFinder program that was developed for Cas9, the off-target effects of Cas9 and ABE are likely to differ, which means that independent off-target assessments will be required in future studies [10, 49].

## Conclusions

In summary, we provide the first report on the application of ABE in large animals to generate sheep with targeted amino acid substitutions. Although we are unable to demonstrate the phenotypes in gene-modified animals at this stage, the results provide an alternative approach to improve animal production, and contribute to the validation of key SNPs that underlie agriculturally important traits.

## Supplementary information

**Additional file 1. Table S1.** sgRNA of target sites. **Table S2.** Oligonucleotides for generating transcription of sgRNA expression vectors. **Table S3.** Primers for genotyping and amplifying Cas9/sgRNA targeted BMPR1B fragment. **Table S4.** Detailed summary of the lambs generated with ABEmax-mediated base editing. **Table S5.** Primers for genotyping and amplifying predicted off-target site fragments. **Table S6.** List of predicted off-target sites.

**Additional file 2: Figure S1.** Overlapping peaks in sequencing maps of the DNA from transfected cells.

**Additional file 3: Figure S2.** Sanger sequencing maps of the DNA from the eight founder animals.

**Additional file 4: Figure S3.** Detection of potential off-targeted sites by Sanger sequencing in founder animals. Five potential off-targeted sites (OT1–OT5) were predicted by Cas-OFFinder. Sanger sequencing was used to determine substitution at predicted target sites for the eight founder animals.

**Additional file 5: Figure S4.** Detection of potential off-targeted sites by Sanger sequencing in sheep fibroblasts. Five potential off-targeted sites (OT1–OT5) were predicted by Cas-OFFinder. Sanger sequencing was used to determine substitutions at predicted target sites in sheep fibroblasts.

## Data Availability

All relevant results are within this paper and its additional files. The raw targeted deep sequencing data is available at NCBI SRA database under the BioProject ID: PRJNA562971.
