# Supplementary material for: Highly efficient generation of sheep with a defined FecBB mutation via adenine base editing
Source: Genet Sel Evol. 2020 Jul 1;52:35. doi: 10.1186/s12711-020-00554-6 (PMC7328262; doi:10.1186/s12711-020-00554-6)
Supplement: Supplementary file 1 — Additional file 1. Table S1. sgRNA of target sites. Table S2. Oligonucleotides for generating transcription of sgRNA expression vectors. Table S3. Primers for genotyping and amplifying Cas9/sgRNA targeted BMPR1B fragment. Table S4. Detailed summary of the lambs generated with ABEmax-mediated base editing. Table S5. Primers for genotyping and amplifying predicted off-target site fragments. Table S6. List of predicted off-target sites. [file 12711_2020_554_MOESM1_ESM.docx]

**Table S1** sgRNA of target sites.

| sgRNA | Targeting site | Location | Strand |
| --- | --- | --- | --- |
| BMPR1B sgRNA | TATATCAGACGGTGTTGATGAGG | Chr6:30695584-30695607 | + |

**Table S2** Oligonucleotides for generating transcription of sgRNA expression vectors.

| Name | Sequence |
| --- | --- |
| BMPR1B sgRNA top strand | ACCGTATATCAGACGGTGTTGATG |
| BMPR1B sgRNA bottom strand | AAACCATCAACACCGTCTGATATA |

**Table S3** Primers for genotyping and amplifying Cas9/sgRNA targeted BMPR1B fragment.

| Name | Sequence | Amplicon (bp) |
| --- | --- | --- |
| BMPR1B_F | AGGTCCAGAGGACGATAGCA | 234 |
| BMPR1B_R | AGGAAACCCTGAACATCGCTAA |  |

**Table S4** Detailed summary of the lambs generated with ABEmax-mediated base editing.

| Recipient sheep | Transferred embryos | Newborns | male parent | female parent |
| --- | --- | --- | --- | --- |
| #640 | 5 | #25 and #34 | #717 | #250 |
| #016 | 4 | #28 | #009 | #654 |
| #132 | 5 | #30 | #001 | #730 |
| #018 | 3 | #31 | #003 | #070 |
| #708 | 5 | #46 | #007 | #172 |
| #608 | 8 | #50 and #52 | #735 | #304 |
| #104 | 6 | No pregnancy | #203 | #370 |
| #100 | 6 | No pregnancy | #203 | #370 |
| #194 | 5 | No pregnancy | #203 | #370 |
| #302 | 5 | No pregnancy | #203 | #370 |
| #002 | 7 | No pregnancy | #735 | #306 |
| #084 | 6 | No pregnancy | #737 | #306 |
| #080 | 5 | No pregnancy | #009 | #654 |
| #064 | 5 | No pregnancy | #003 | #070 |
| #292 | 6 | No pregnancy | #003 | #070 |
| #632 | 6 | No pregnancy | #713 | #036 |
| #022 | 5 | No pregnancy | #731 | #218 |
| #908 | 5 | No pregnancy | #731 | #218 |

**Table S5** Primers for genotyping and amplifying predicted off-target site fragments.

| Off-target site | Sequence (5'-3') | Amplicon (bp) |
| --- | --- | --- |
| BMPR1B -OT1F | ACTCGACTTTTCTATCTGCAT | 294bp |
| BMPR1B -OT1R | GTATTGGCACCACTATCACCA |  |
| BMPR1B -OT2F | TCCTCCAGTTACTTGGCATAC | 472bp |
| BMPR1B -OT2R | CCAAGACAAATTTCTAGCAGT |  |
| BMPR1B -OT3F | GTATTGGGTATGAAACGGAGG | 397bp |
| BMPR1B -OT3R | CTAAATGAGCCCAAATCTCCT |  |
| BMPR1B -OT4F | AGATGATTCTTTCGAAGGCAA | 319bp |
| BMPR1B -OT4R | AACAGCACAGATTAATGGTCA |  |
| BMPR1B -OT5F | GACCCTACAGCTTTGCATGG | 340bp |
| BMPR1B -OT5R | GAATGGCTGCTCTCTAGCTCT |  |

**Table S6** List of predicted off-target sites.

| Position | 20 | 19 | 18 | 17 | 16 | 15 | 14 | 13 | 12 | 11 | 10 | 9 | 8 | 7 | 6 | 5 | 4 | 3 | 2 | 1 | N | G | G | Location | | | |
| --- | --- | --- | --- | --- | --- | --- | --- | --- | --- | --- | --- | --- | --- | --- | --- | --- | --- | --- | --- | --- | --- | --- | --- | --- | --- | --- | --- |
| sgRNA | T | A | T | A | T | C | A | G | A | C | G | G | T | G | T | T | G | A | T | G | A | G | G | Chr. | Start | End | Strand |
| OT1 | T | A | T | T | T | C | A | G | A | A | G | G | T | T | T | T | G | A | T | G | A | G | G | 12 | 23,285,473 | 23,285,495 | + |
| OT2 | T | A | T | A | T | C | A | C | A | C | T | G | T | G | T | T | G | C | T | G | A | G | G | 2 | 204,015,205 | 204,015,227 | + |
| OT3 | T | A | T | A | T | T | A | G | A | T | G | C | T | G | T | T | G | A | T | G | T | G | G | 1 | 41,982,993 | 41,983,015 | + |
| OT4 | T | A | T | T | T | C | A | G | A | C | A | G | T | G | T | T | C | A | T | G | A | G | G | 1 | 89,475,237 | 89,475,259 | – |
| OT5 | C | A | T | A | T | C | A | G | A | G | G | G | T | G | G | T | G | A | T | G | A | G | G | 15 | 41,881,083 | 41,881,105 | – |
