# Supplementary figures and images for: Highly efficient generation of sheep with a defined FecBB mutation via adenine base editing
Source: Genet Sel Evol. 2020 Jul 1;52:35. doi: 10.1186/s12711-020-00554-6 (PMC7328262; doi:10.1186/s12711-020-00554-6)

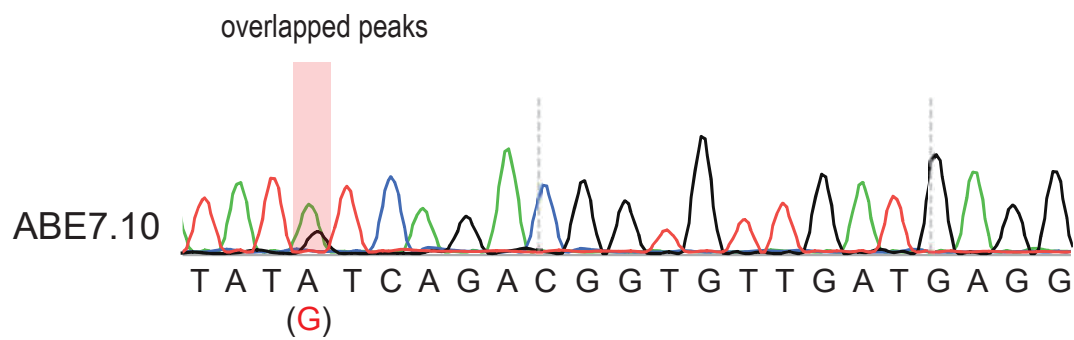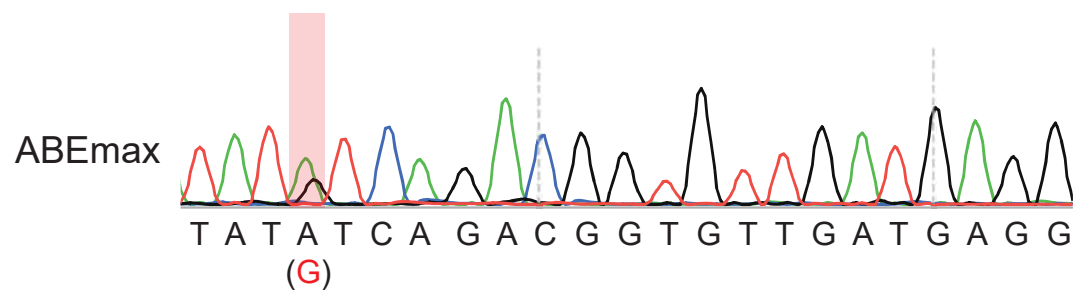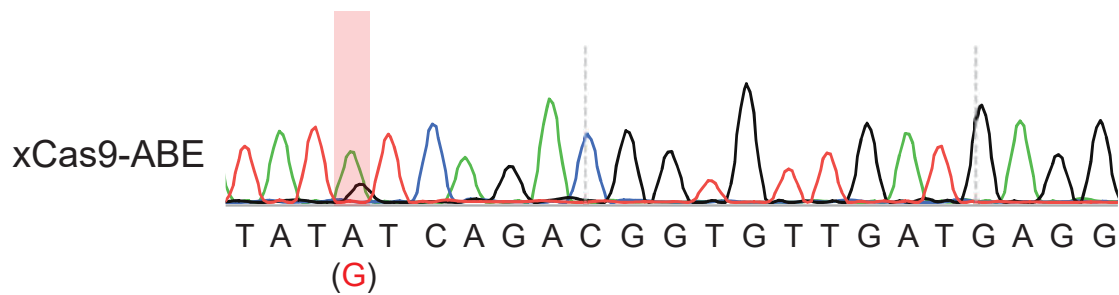

Supplement: Supplementary file 2 — Additional file 2: Figure S1. Overlapping peaks in sequencing maps of the DNA from transfected cells. [file 12711_2020_554_MOESM2_ESM.pdf]

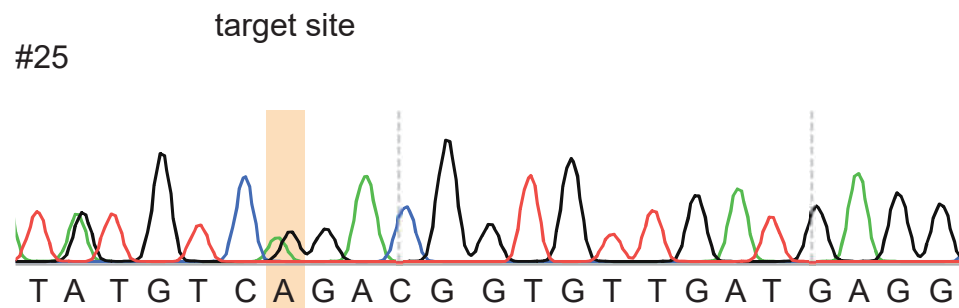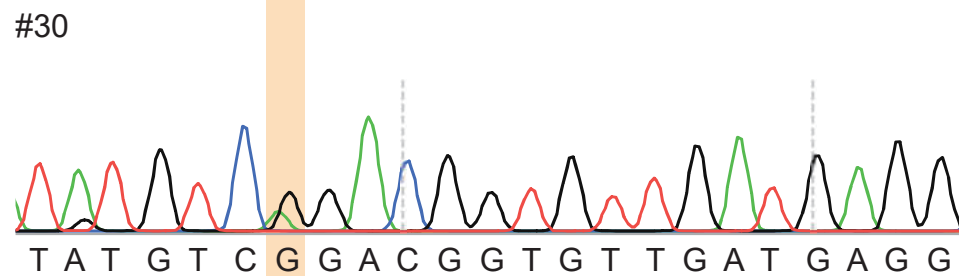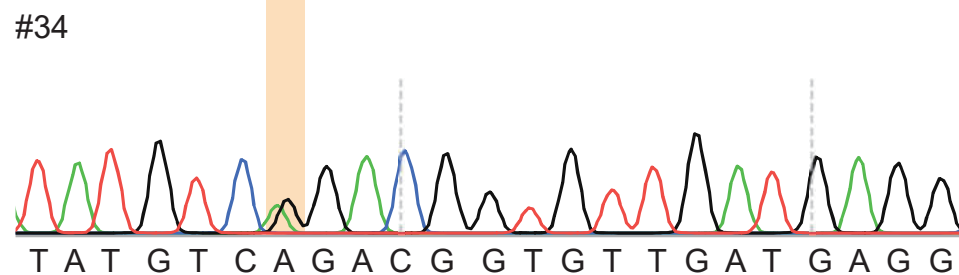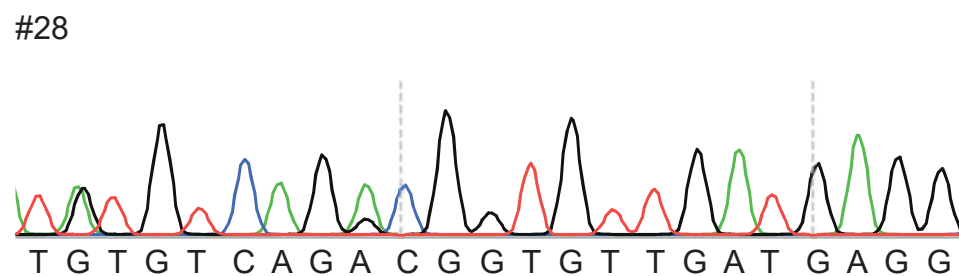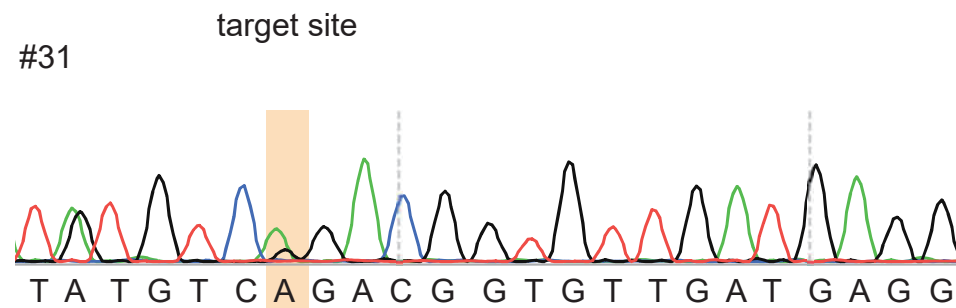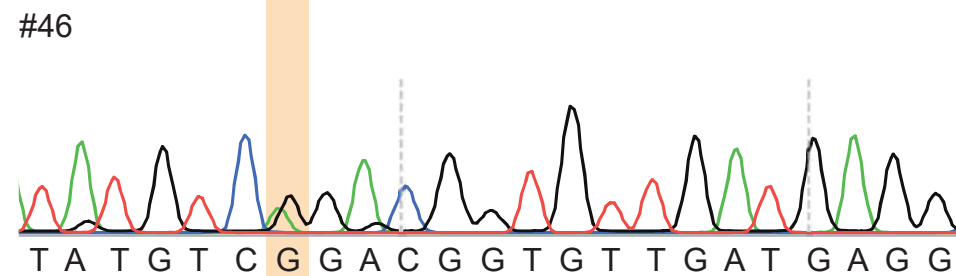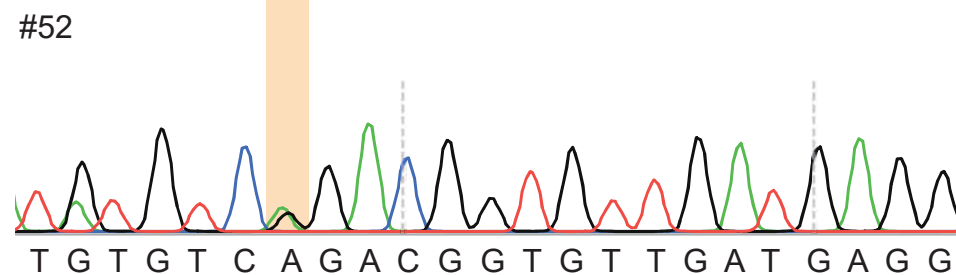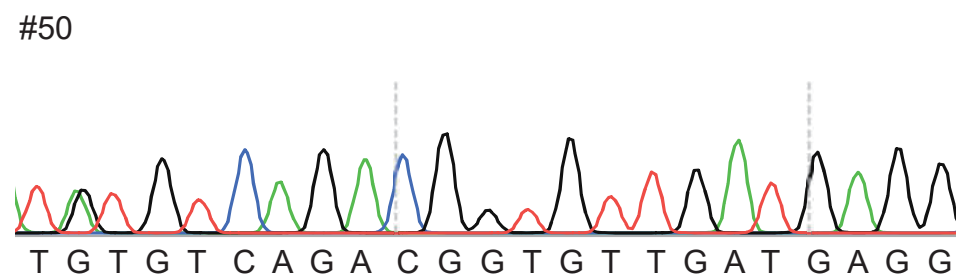

Supplement: Supplementary file 3 — Additional file 3: Figure S2. Sanger sequencing maps of the DNA from the eight founder animals. [file 12711_2020_554_MOESM3_ESM.pdf]

OT1

OT2

OT3

OT4

OT5

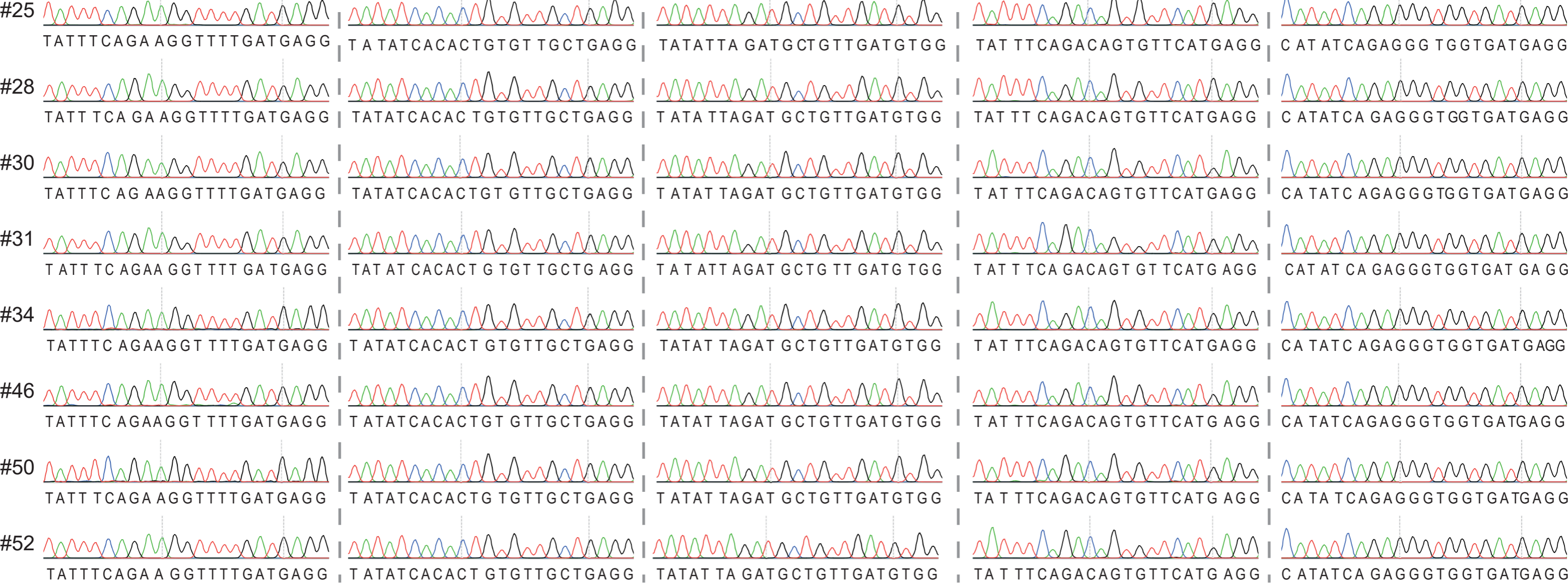

Supplement: Supplementary file 4 — Additional file 4: Figure S3. Detection of potential off-targeted sites by Sanger sequencing in founder animals. Five potential off-targeted sites (OT1–OT5) were predicted by Cas-OFFinder. Sanger sequencing was used to determine substitution at predicted target sites for the eight founder animals. [file 12711_2020_554_MOESM4_ESM.pdf]

## sheep fetal fibroblasts

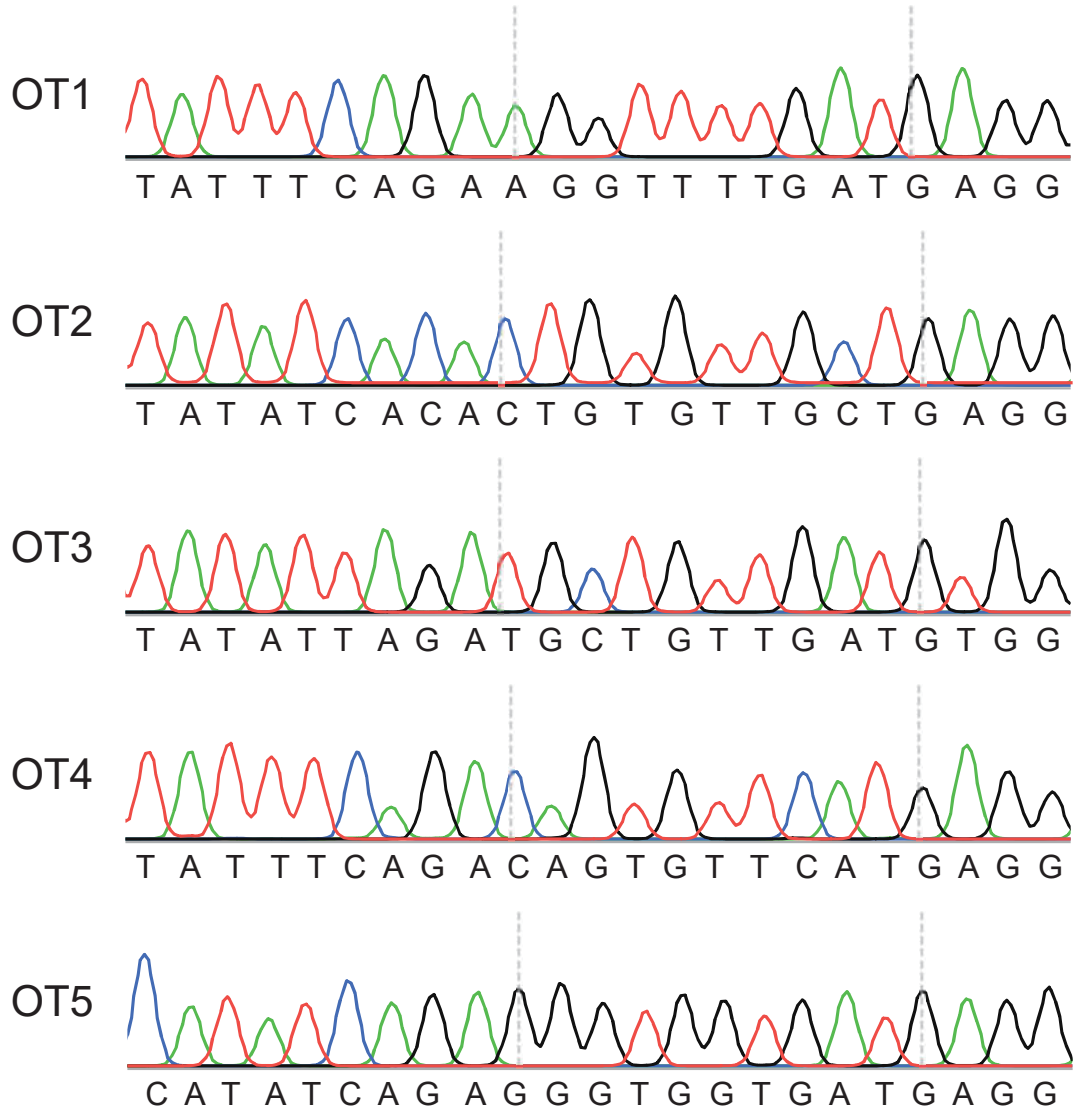

Supplement: Supplementary file 5 — Additional file 5: Figure S4. Detection of potential off-targeted sites by Sanger sequencing in sheep fibroblasts. Five potential off-targeted sites (OT1–OT5) were predicted by Cas-OFFinder. Sanger sequencing was used to determine substitutions at predicted target sites in sheep fibroblasts. [file 12711_2020_554_MOESM5_ESM.pdf]
